# Case Report: Diagnostic challenge between epileptic seizure and tetany in Albright hereditary osteodystrophy

**DOI:** 10.3389/fradi.2026.1753367

**Published:** 2026-05-29

**Authors:** Madeleine D. Burns, Madelynn Paul, Alekya Menta, Olivia Hofheinz, Sachin Vallamkonda, Rebecca E. Irvine, Alexis P. Kruger, Eric D. Schwartz, Anna D. Hohler

**Affiliations:** 1 Boston University Chobanian & Avedisian School of Medicine, Boston, MA, United States; 2 Department of Neurology, Boston Medical Center Brighton, Brighton, MA, United States; 3 Tufts University School of Medicine, Boston, MA, United States; 4 Faculty of Medicine, Imperial College London, London, England, United Kingdom; 5 Department of Radiology, Boston Medical Center Brighton, Brighton, MA, United States

**Keywords:** Albright hereditary osteodystrophy, case report, electrolyte abnormalities, seizures, tetany

## Abstract

**Background:**

Albright hereditary osteodystrophy (AHO) is an epigenetically inherited condition resulting in end-organ resistance to parathyroid hormone (PTH) and consequent hypocalcemia, leading to tetany and seizures. AHO-driven calcification of the basal ganglia can often be visualized on computed tomography (CT).

**Case description:**

We report the case of a 36-year-old patient with AHO presenting with epileptic seizure vs. tetany, with an analysis of non-contrast head CT. An adult patient presented with bilateral clenching of fists, progressing to motor weakness, atonic loss of upright posture, and diffuse clonic shaking. The patient's history included alcohol use disorder with generalized tonic–clonic seizures on withdrawal. A physical examination revealed short stature, round facies, short fourth metacarpal bones, and subcutaneous calcifications on the right extremities. Laboratory tests showed leukocytosis with neutrophilic predominance, hypokalemia, hypocalcemia, hyperphosphatemia, and elevated PTH. A non-contrast head CT showed marked bilateral basal ganglia calcifications. An EEG showed generalized slowing with no epileptiform activity. This presentation is consistent with tetany secondary to poor medication adherence in the setting of AHO.

**Conclusions:**

Tetany in the setting of AHO may mimic epileptic seizure because of hypocalcemia causing clonus. Home medication adherence, early disease diagnosis, and prompt electrolyte correction are critical to the management of AHO.

## Introduction

Albright hereditary osteodystrophy (AHO), also known as pseudohypoparathyroidism Type 1a, is a rare autosomal-dominant condition that results from a mutation on the maternal allele of the GNAS gene (1, 2). Currently, data on AHO are limited, but disease prevalence is estimated to be approximately 1 in 20,000 (1, 3). GNAS is subject to genomic imprinting, where gene expression depends on parent-specific silencing mechanisms (1, 2, 4). This gene encodes the Gs alpha subunit and, when mutated, results in end-organ resistance to the parathyroid hormone (PTH), particularly at the renal tubule. This causes electrolyte abnormalities such as severe hypocalcemia, hyperphosphatemia, and secondary hyperparathyroidism (1–3). AHO is also associated with resistance to other hormones, including thyroid-stimulating hormone, growth hormone–releasing hormone, luteinizing hormone, follicle-stimulating hormone, and calcitonin, due to shared reliance on Gs alpha–mediated cyclic adenosine monophosphate pathways (1, 3).

Patients typically present in early childhood with symptoms of hypocalcemia such as tetany, jitteriness, paresthesia, perioral numbness, facial twitching on percussion over the course of the facial nerve (Chvostek sign), carpopedal spasm on inflating sphygmomanometer cuff (Trousseau's sign), and, in severe cases, epileptic seizures (1, 2, 5). Typical physical features of AHO are short stature, round facies, dental anomalies, moderate intellectual disability, relative macrocephaly, brachydactyly in the fourth and fifth metacarpals with dimpling over the knuckles, hyperparathyroid bone disease, metabolic syndrome, and early-onset obesity (1, 6). On imaging, basal ganglia calcification is commonly visualized and, in more severe cases, subcortical white matter calcification can occur (1, 3, 5, 7, 8).

Herein, we report an adult patient with Albright hereditary osteodystrophy whose presentation was concerning for epileptic seizure vs. tetany.

## Case description

A 36-year-old woman was admitted to the emergency department by emergency medical services for evaluation of suspected, witnessed epileptic seizure vs. tetany. The event began with acute bilateral clenching of the fists and then progressed to weakness, atonic loss of upright posture, and motor control, followed by diffuse clonic shaking. The patient was unable to speak during the event but maintained consciousness. Upon presentation to the emergency department, she was found to be hypertensive and unable to unclench her fists bilaterally. The patient endorsed a recent heavy alcohol intake of 4–5 pints of vodka daily (the last drink was the night prior to presentation), as well as non-adherence to home medications such as calcitriol 0.5 mcg daily and vitamin D3. Her medical history was significant for tetany secondary to pseudohypoparathyroidism in the setting of a known diagnosis of Albright hereditary osteodystrophy (GNAS testing performed at an outside hospital), alcohol use disorder with a history of complicated withdrawal including generalized tonic–clonic seizures (the most recent being three months prior to presentation), heart failure with reduced ejection fraction (27% EF on echocardiogram performed two and a half years prior to presentation), and primary hypertension. A family history of Albright hereditary osteodystrophy was unknown. The neurologic examination was without focal deficits, except for rigid bilateral upper extremities with carpopedal spasms, moderately improved with 2 mg lorazepam (ativan), and a mild intention tremor on finger-to-nose testing. Chvostek and Trousseau signs were not evaluated. The remainder of the physical examination revealed short stature (1.52 meters), round facies, short fourth metacarpal bones bilaterally, and subcutaneous calcifications on the right upper extremity, right lower extremity, and right foot. Moreover, a developmental delay was endorsed by the patient. Notable laboratory test results are summarized in Table 1. A non-contrast computed tomography (CT) of the head was notable for marked bilateral basal ganglia calcifications along with subcutaneous skull and scalp calcifications, stable from prior imaging at outside hospital evaluations. Routine EEG was negative for epileptiform activity but did show generalized slowing suggestive of mild encephalopathy.

**Table 1:** Relevant laboratory values with reference ranges.

Laboratory test	Value	Reference range
White blood cells	11.8	4.0–11.0 × 10^9^/L
Potassium	2.5	3.5–5.0 mmol/L
Calcium	5.4	8.6–10.2 mg/dL
Phosphorus	4.8	2.5–4.5 mg/dL
Magnesium	1.9	1.7–2.2 mg/dL
Creatine kinase	705	30–150 U/L
Vitamin D	21	≥30 ng/mL
Parathyroid hormone	378	10–65 pg/mL
Serum ethanol	<10	<10 mg/dL
Urine toxicology	Negative	N/A

Upon serial neurology evaluation, the patient’s presentation was found to be most likely tetany in the setting of severe hypocalcemia secondary to poor outpatient medication adherence for AHO. She was started on the Clinical Institute Withdrawal Assessment Alcohol protocol with a chlordiazepoxide sliding scale, and her electrolytes were replenished, including potassium, calcium, and magnesium. The patient was discharged home on her outpatient medications that included calcitriol 0.5 mcg QD and vitamin D3.

## Discussion

This report describes an adult patient with Albright hereditary osteodystrophy presenting with concern for epileptic seizure vs. tetany. Albright et al. were the first to describe PTH resistance with severe hypocalcemia, hyperphosphatemia, and epileptic seizures in the year 1942 (6). Epileptic seizures in AHO are believed to result from calcification of specific brain structures, particularly the basal ganglia, which is most commonly affected (2, 5). In rare and more severe cases, calcification extends to the subcortical white matter, which has been linked to epileptic seizures as well as neuropsychiatric disorders, movement disorders, and Parkinsonism (2, 5, 9, 10).

Intracranial calcifications have been associated with severe neurodevelopmental outcomes including epilepsy in other neurologic disorders such as Fahr syndrome, as well as in congenital viral infections such as cytomegalovirus, Zika virus, and lymphocytic choriomeningitis virus (11, 12). Fahr syndrome is a neurologic disorder characterized radiographically by calcium deposits in cerebral structures including the basal ganglia, cerebellar dentate nuclei, and cerebral cortical white matter secondary to hypoparathyroidism (12). Although Fahr syndrome shares many of the same clinical and radiographic manifestations of hypocalcemia as AHO, including movement disorders and psychiatric conditions, it is unique to AHO with rare reports of thrombocytopenia, which has implications for intracranial hemorrhage secondary to recurrent seizures in this at-risk population (12).

In our patient, a non-contrast head CT imaging showed increased signal hyperdensity in the basal ganglia, suggestive of bilateral calcification of this structure (Figure 1). In addition, multiple supracranial focal calcifications were seen on the non-contrast CT head, consistent with the subcutaneous ossification discussed previously (Figure 2). These imaging findings align with prior reports of brain parenchymal and soft tissue calcification in AHO, which made CT imaging sufficient for radiographic evaluation in this case over MRI (2, 5, 8). Given these abnormal imaging findings and the patient's hypocalcemia, epileptic seizures secondary to structural vs. metabolic abnormalities such as AHO-related calcifications and Fahr Syndrome were initially considered. However, the patient's preserved awareness during the episode and a negative EEG during admission reduced the likelihood of epileptic seizure as the cause of her current presentation. Furthermore, the calcifications seen on CT did not show interval change from prior evaluations outside the hospital, making epileptic seizures secondary to cerebral calcifications in AHO less likely. Alcohol withdrawal seizures were also considered given her history of complicated withdrawal but were determined to be less likely given the short duration between her last drink and initiation of her symptoms. Consequently, tetany secondary electrolyte imbalance in the setting of medication non-adherence emerged as the more likely diagnosis.

**Figure 1:**
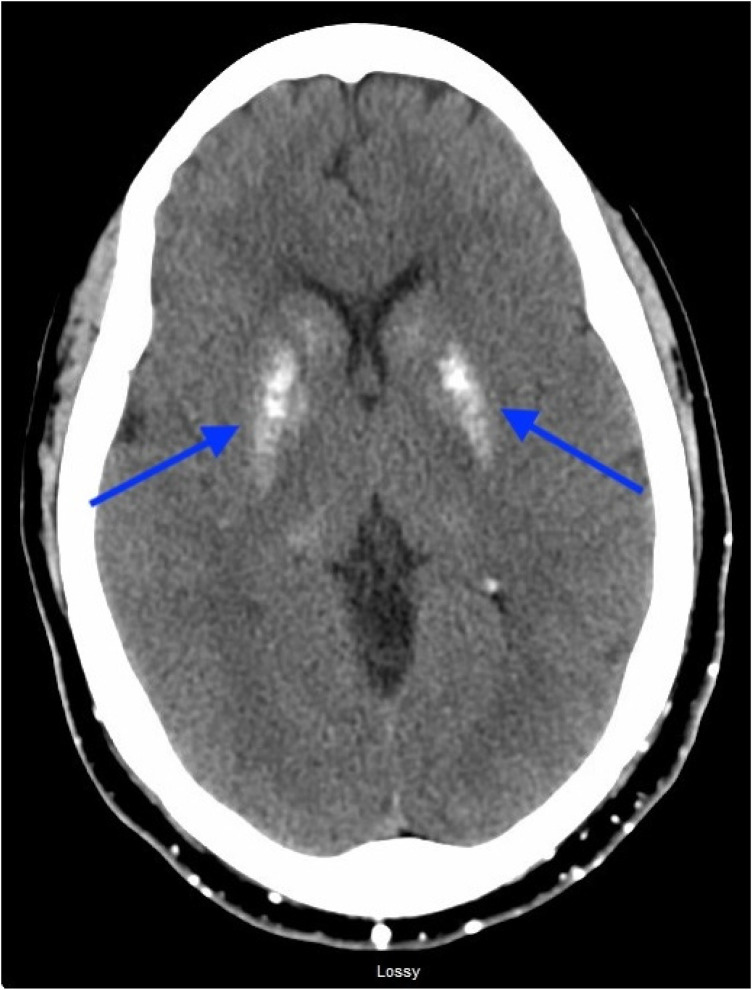
A non-contrast CT head showing hyperdensity in the bilateral basal ganglia (blue arrows), suggestive of calcification of these structures.

**Figure 2:**
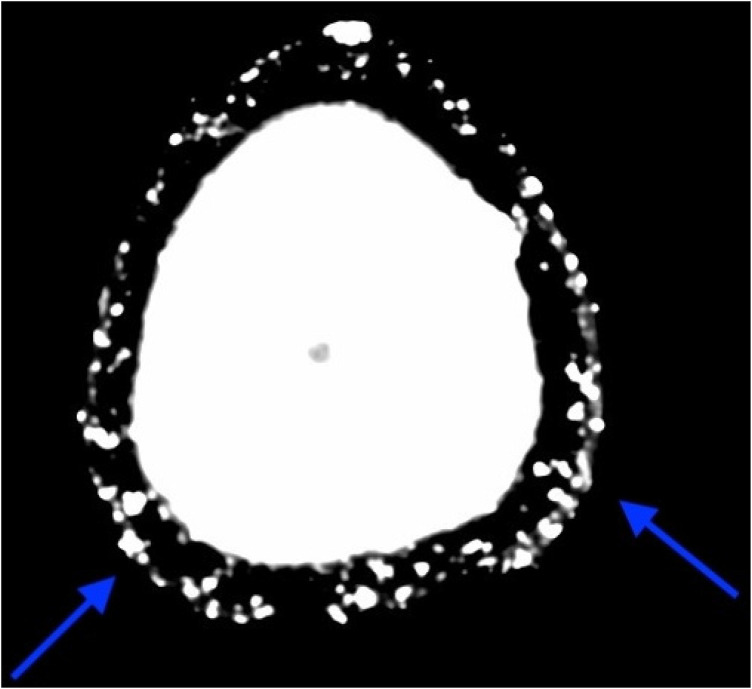
A non-contrast CT head showing multiple supracranial focal calcifications [blue arrows], consistent with the subcutaneous ossification seen in AHO.

AHO can mimic epileptic seizures, as seen in our patient, which can ultimately be a presentation of tetany (5). In the setting of hypocalcemia, individuals may experience full-body involvement with clonus. It is important to assess for home medication adherence in AHO, as electrolyte imbalance plays a central role in symptomatology. Early diagnosis of AHO and prompt correction of electrolyte abnormalities are critical. Multidisciplinary management and regular outpatient follow-up to support medication adherence are recommended, given the multisystem nature of the disorder.

## Conclusion

In conclusion, this case contributes to the current literature by detailing the clinical symptoms, radiographic findings, diagnostic workup, and management of an adult with AHO who presented with tetany in the setting of severe hypocalcemia, likely secondary to poor medication adherence. Overall, it highlights the importance of maintaining proper electrolyte balance in individuals with AHO.

## Data Availability

The original contributions presented in the study are included in the article/Supplementary Material, and further inquiries can be directed to the corresponding author/s.
